# Cryogenic transmission electron microscopy: the technique of choice for the characterization of polymeric nanocarriers

**DOI:** 10.1186/s13550-017-0290-3

**Published:** 2017-05-22

**Authors:** Adrianus C. Laan, Antonia G. Denkova

**Affiliations:** Department of Radiation Science and Technology, Delft University of Technology, Delft, the Netherlands

## Abstract

This letter is meant to make scientists aware of the proper application of transmission electron microscopy (TEM) for the assessment of polymeric self-assemblies. Cryogenic (cryo)-TEM should be the method of choice. Here, we show the difference in morphologies observed in the same sample when using cryo-TEM and when using TEM with drying, demonstrating the importance of choosing the proper method.

## Correspondence

Dear Editor,

In our article published on February 2016, we have reported a method for radiolabeling polystyrene-b-poly(ethylene oxide) (PS-b-PEO) diblock copolymer micelles [1]. As part of the physical characterization of the produced nanocarriers, we have done analysis with transmission electron microscopy (TEM) using the drying method. However, it was brought to our attention that this characterization method involving drying is not ideal for the investigation of the morphologies of self-assemblies, even when these assemblies are composed of block copolymers having extremely slow-exchange kinetics. Cryo-TEM is the technique that should be used for the assessment of soft-matter assemblies, as pointed out in the critical study carried out by Stuart et al. [2]. The major cause of discrepancies between the drying method and cryo-TEM is related to the preparation procedure of the samples on the TEM grid. With the drying method, a drop of the sample is dried onto a grid by evaporation of the solvent from the sample, which can result in deformation or even complete destruction of the particles. In the case of cryo-TEM, the sample is rapidly frozen on the grid, i.e., instantly vitrified, impairing any changes of the morphology of the particles [2–5].

**Fig. 1:**
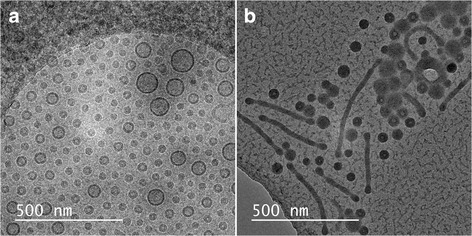
Micrographs of a sample of polymeric self-assemblies composed of PS-b-PEO 9500-18000 at a concentration of 10 mg/mL. **a** Micrograph obtained with cryo-TEM. **b** Micrograph obtained with TEM using drying

Consequently, we decided to carry out cryo-TEM analysis of the ‘micelles’ that we previously observed using the drying method. This analysis showed us that besides micelles, vesicles are also formed with sizes similar to the micelles but also larger ones (see Fig. 1a). For direct comparison, we have analysed the same sample again using the drying method (see Fig. 1b). Clearly, the sample prepared with the drying method does not exhibit any vesicles, while these species are clearly visible in the cryo-TEM images. These findings are in line with literature reports indicating that some type of self-assemblies and morphologies can be destroyed during the preparation procedure when drying is involved [2–5]. This comparison also shows an additional drawback of using the drying method, i.e., the aggregation of particles, impairing the detection of individual entities.

In regard to our previous publication [1], this means that the size range of the nano-assemblies is somewhat different from what we have reported based on the TEM analysis. On the other hand, the results obtained by dynamic light scattering, as reported, do give a good indication on the actual size range and polydispersity of the self-assemblies. We believe that our new findings do not affect the conclusions on the radiolabeling of the polymeric carriers, as this mechanism remains unchanged. This mechanism is based on the loading of the radionuclide in the hydrophobic compartment of the nanocarriers by employing a lipophilic ligand to complex the metal. In the case of the micelles, the PS core was radiolabelled, and in the case of the vesicles, the hydrophobic part of the bi-layered membrane was used to retain the radiolabel.

With this letter, we would like to emphasize that with the more suitable technique, cryo-TEM, we were able to get better understanding on our method and the produced self-assemblies. Furthermore, with this letter, we hope to help other researchers to realize that TEM using the drying method as well as scanning electron microscopy or atomic force microscopy are not appropriate techniques for determining the morphology of any soft-matter species [2–4].

## Materials and methods

For preparation of the samples: in a 20 mL glass vial, 100 μL of a solution of PS-b-PEO 9500-18000 block copolymer in chloroform was added to 2.3 mL of 10 mM HEPES buffer pH 7.4 to reach the final polymer concentration of 10 mg/mL. The mixture was stirred at room temperature in a fume hood using a glass stirring bar in a vial without cap for about 2 h until the chloroform had evaporated.

The JEOL JEM-1400Plus, 120 kV transmission electron microscope with a LaB6 emitter, was used for the analysis.

For the drying method: directly before analysis, 5 μL of sample was put onto a hydrophilized TEM grid (Quantifoil “R1.2/1.3” holey carbon film on Cu 200 mesh), the excess of the sample was removed with filter paper, and the grid was left to dry for about 1 min before placing the grid, using a sample holder, in the microscope for TEM analysis.

For cryo-TEM, the Leica EM GP automatic plunge freezer was used to prepare the sample on the TEM grid. The blotting chamber was kept at 20 °C and >90% RH, 5 μL of sample was put onto the hydrophilized grid, and the grid was blotted (one-sided front side blotting) with filter paper for 4 s before plunging in liquid ethane. The grid was transferred to the cryo sample holder and inserted in the microscope.
